# CircNetVis: an interactive web application for visualizing interaction networks of circular RNAs

**DOI:** 10.1186/s12859-024-05646-4

**Published:** 2024-01-17

**Authors:** Thi-Hau Nguyen, Ha-Nam Nguyen, Trung Nghia Vu

**Affiliations:** 1 University of Engineering and Technology, Vietnam National University in Hanoi, Hanoi, 84024 Vietnam; 2 Department of Information Technology, Electric Power University, Hanoi, 84024 Vietnam; 3 Department of Medical Epidemiology and Biostatistics, Karolinska Institutet, 17177 Stockholm, Sweden

**Keywords:** Circular RNA, Interaction network, Visualization

## Abstract

Analyzing the interactions of circular RNAs (circRNAs) is a crucial step in understanding their functional impacts. While there are numerous visualization tools available for investigating circRNA interaction networks, these tools are typically limited to known circRNAs from specific databases. Moreover, these existing tools usually require complex installation procedures which can be time-consuming and challenging for users. There is a lack of a user-friendly web application that facilitates interactive exploration and visualization of circRNA interaction networks. CircNetVis is an interactive online web application to enhance the analysis of human/mouse circRNA interactions. The tool allows three different input formats of circRNAs including circRNA IDs from CircBase, circRNA coordinates (chromosome, start position, end position), and circRNA sequences in the FASTA format. It integrates multiple interaction networks for visualization and investigation of the interplay between circRNA, microRNAs, mRNAs and RNA binding proteins. CircNetVis also enables users to interactively explore the interactions of unknown circRNAs which are not reported from previous databases. The tool can generate interactive plots and allows users to save results as output files for offline usage. CircNetVis is implemented as a web application using R-shiny and freely available for academic use at https://www.meb.ki.se/shiny/truvu/CircNetVis/.

**Supplementary Information:**

The online version contains supplementary material available at 10.1186/s12859-024-05646-4.

## Introduction

Circular RNAs (circRNAs) are a distinct class of single-stranded non-coding RNA molecules that are covalently linked in a closed-loop structure. CircRNAs exhibit diverse biological functions within cells through their interactions with other molecules [1]. For example, a circRNA can function as a competing endogenous RNA (ceRNA) by acting as a sponge to bind to microRNAs (miRNAs) and regulate their function [2]. Since miRNAs can play a role in post-transcriptional expression of target genes, the circRNA is able to indirectly regulate the gene expression [1, 3]. Additionally, circRNAs can affect gene expression by interacting with RNA binding proteins (RBPs) and modulating mRNA stability [4]. Some recent studies have demonstrated that circRNAs not only serve as molecular markers but also participate in cancer proliferation and invasion by regulating miRNAs in various types of cancer. This regulation can occur through circRNA-miRNA-mRNA regulatory network [5, 6] or circRNA-RBP interaction network [7].

A number of tools have been developed to support the discovery of biological functions of circRNAs through interaction network analysis, for example, circlncRNAnet [8], RAID [9], circPlant [10], CircMiMi [11], Circr [12]. However, most of the existing tools primarily focus on circRNAs provided by specific circRNA databases such as circBase [13], which limits their applicability for studying novel circRNAs. Of note, the circRNA databases are mainly constructed using data from previous studies and may not encompass all potential circRNAs in newly generated data. Furthermore, these tools often require experienced users to download, configure and install the software, which can be time-consuming and challenging. Finally, most of the tools lack an interactive interface that would facilitate the exploration and analysis of circRNA interaction networks.

Therefore, we have developed CircNetVis, an online web application designed to facilitate the visualization and analysis of circRNA interaction networks. CircNetVis enables users to visualize and export the results of different types of interactions including circRNA-miRNA, miRNA-mRNA, circRNA-RBP from one or multiple (unknown) circRNAs of interest. For circRNA-miRNA, three commonly-used interaction predictors including TargetScan [14], RNAhybrid [15] and miRanda [16] are utilized. CircNetVis facilitates the investigation of interaction network by allowing users to input miRNAs and genes of interest and apply different filters for the interaction predictors. CircNetVis is implemented using R-shiny with external tools running in a Linux environment, and available at https://www.meb.ki.se/shiny/truvu/CircNetVis/. The source codes and data resources to run the web application are provided in its GitHub page at https://github.com/nghiavtr/CircNetVis.

## Implementation

CircNetVis utilizes R-shiny [17] to build the web interface (frontend) with all calculations (backend) running on a Linux environment. The current version of this application supports only circRNAs detected in the human genome reference hg19 and Mus musculus (mouse) genome reference mm9. The details of methodologies, tools and their parameters are provided in the web application. A brief comparison of CircNetVis with existing tools are provided in Table 1.

**Table 1:** Comparison between CircNetVis and existing tools

Method	CircNetVis	circlncRNAnet [8]	RAID [9]	circPlant [10]	CircMiMi [11]	Circr [12]
Can work with new circRNA	Yes	No	No	Yes	No	No
Allow circRNA-sequence input	Yes	No	No	Yes	No	Yes
Species	Human/mouse	Human	Bacteria, fungi, insects, nematodes, plants, vertebrates, viruses	Plant	18 species (16 animals and 2 plants)	Human/mouse
Online/offline	Online and offline	Online	Online	Offline	Online and offline	Offline
circRNA—miRNA interaction prediction	Yes	Yes	Yes	Yes	Yes	Yes
circRNA—miRNA—mRNA network construction	Yes	Yes	No	Yes	Yes	No
circRNA-RNA binding proteins interaction	Yes	Yes	Yes	No	No	No
Pathway analysis	Yes	Yes	No	No	No	No
circRNA-miRNA interaction predictors	miRanda, RNAhybrid, TargetScan	miRanda, RNAhybrid, TarPmiR	EIMMo, miRanda, miRDB, TargetScan	TargetFinder, Tapir	miRDB, miRTarBase, MirTarget	miRanda, RNAhybrid, TargetScan

### Backend description

The overview of the backend of the CircNetVis web application is presented in Additional file 1: Figure S1. In the backend of CircNetVis, the following key public databases are collected: circBase [13] for annotated circRNAs, miRbase [18] for miRNAs, CircInteractome database [19] for circRNA-RBP interactions, TargetScan database [14] version 72 for miRNA-mRNA interactions. To identify circRNA-miRNA interactions, three widely used prediction tools are utilized including miRanda [16] version v3.3a, RNAhybrid [15] version 2.1.2, and TargetScan [14] version 72. These tools get input from the pseudo-sequences of circRNAs which are generated using Circall-simulator [20]. The further detailed generation of pseudo-sequences of circRNAs are provided in the Additional file 1. For annotated circRNAs from circBase, a miRNA-mRNA interaction database is built in advance to speed up the processing. For novel circRNAs not found in circBase, the prediction tools will be performed on-the-fly during the use of CircNetVis.

### Frontend description

**Fig. 1:**
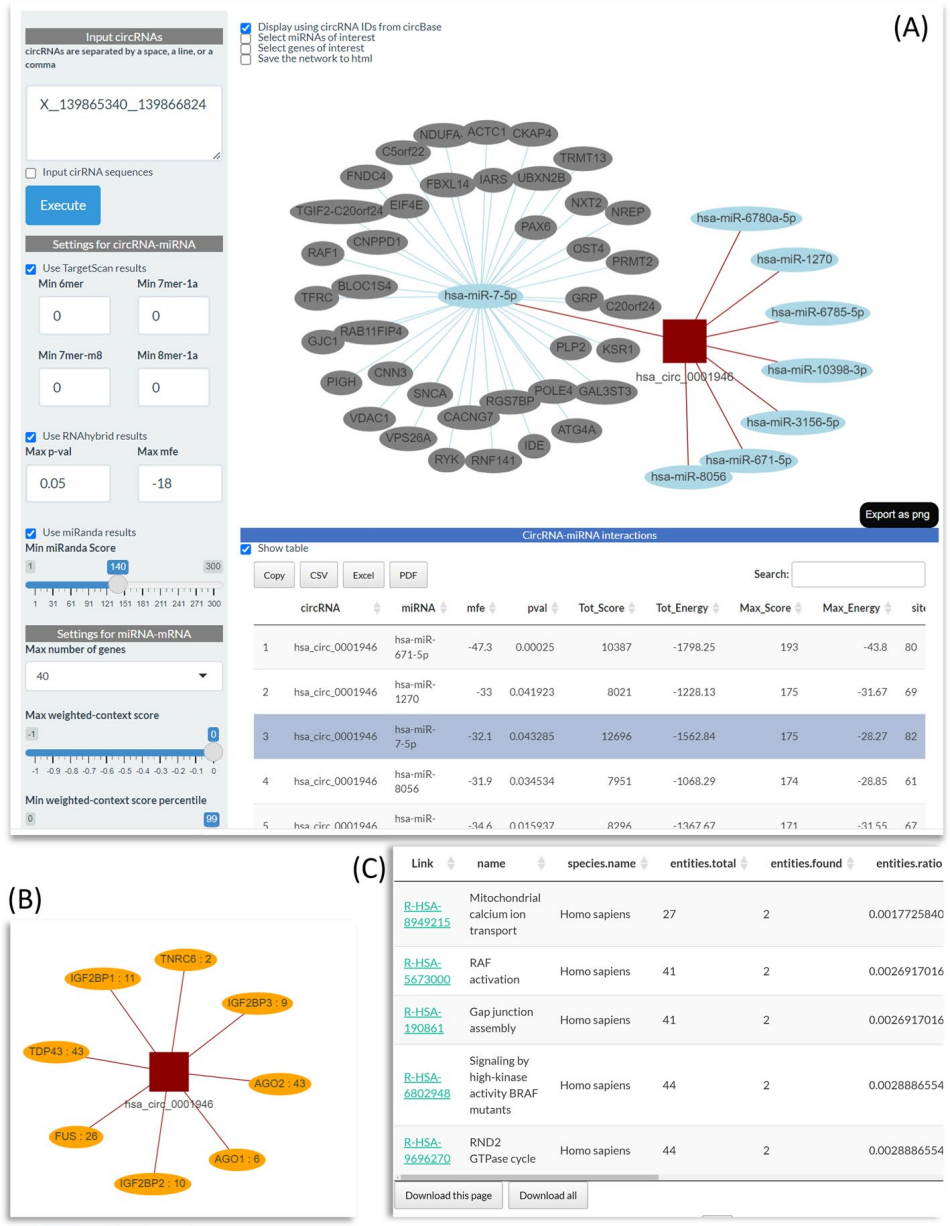
Example of use of CircNetVis for investigating interaction networks of hsa_circ_0001946 (or X__139865340__139866824). **A** Control panel and results of circRNA-miRNA-mRNA interactions. **B** Visualization of circRNA-RBP interaction. **C** Pathway analysis of genes reported in the circRNA-miRNA-mRNA network

In the frontend, CircNetVis takes input from a single or multiple circRNAs of interest, Fig. 1A. The input circRNAs can be provided in three different types of format. First, users can use directly the circRNA.ID from circBase, for example, hsa_circ_0001946. Second, the circRNA information can be provided as the format of normID in Circall [20] (of note, circRNA can be predicted from other circRNA prediction tools, however for the purpose of consistency, the circRNA IDs need to be re-formatted as the normID). A normID includes a chromosome name (chr), a start position and an end position (in the one-based coordinate system of the genome reference) concatenated together as chr__start__end, for example X__139865340__139866824. Finally, CircNetVis also allows the input from the sequences of the circRNAs in the standard FASTA format.

The exploration of circRNA-miRNA interactions can be controlled by the filters specific to each of the three interaction predictors in the setting control panel, Fig. 1A. Users can use one or combine multiple predictors to obtain confident interactions. The default thresholds for the filters are often used in previous studies of circRNA-miRNA-mRNA networks [5, 6]. These values are easily adjustable by users. In addition, users can limit the results by inputting the list of miRNAs and genes of interest. These miRNAs and genes can be derived from specific analyses, such as differential expression analysis of a research project. This function enhances the flexibility of CircNetVis for application in different projects.

The circRNA-miRNA-mRNA interaction network is visualized using a flexible interactive network visualization R-package, visNetwork version 2.1.0 [21]. visNetwork allows users to move nodes, change edge position, and interact with the network easily. The data of the circRNA-miRNA and miRNA-mRNA interactions are reported in tables for further investigation or exporting to files.

In addition, CircNetVis provides a separate circRNA-RBP interaction network for the circRNAs of interest collected from the CircInteractome database which was previously built using CLIP-seq data [19], Fig. 1B. CircNetVis also utilizes the gene set enrichment analysis of Reactome [22] to suggest biological pathways relevant to the genes in the circRNA-miRNA-mRNA network, Fig. 1C.

## Application

To illustrate how CircNetVis works, in Fig. 1, we present a case study of *hsa_circ_0001946*, a circRNA of *CDR1as* gene, which has been extensively studied in the literature [23].

The obtained circRNA-miRNA network reports eight miRNAs passing the default filters of the three circRNA-miRNA prediction tools. Among those, the interaction of *hsa-miR-7-5p* with *hsa_circ_0001946* is well validated in human [23]. This interaction, which is highlighted in the table at the bottom of Fig. 1A, shows strong signals with a high number of binding sites of 8mer1a, 7merm8, 7mer1a and 6mer reported by TargetScan, a high miRanda score (Max score = 175) and a low RNAhybrid mfe (minimum of free energy) of -32.1. Multiple mRNAs may interact with this miRNA, as illustrated in the figure. Among these interactions, the association between *hsa-miR-7-5p* and paired box gene 6 (*PAX6*) has been investigated in the literature [24]. Two other top interactions of *hsa_circ_0001946* with miRNAs (ranked by Max_Score of miRanda) are also previously validated, including *hsa-miR-1270* [25] and *hsa-miR-671-5p* [26]. Regarding circRNA-RBP, CircNetVis provides a visualization of the corresponding interactions from CircInteractome database, Fig. 1B. AGO2 is one of RBPs densely bounding around the circRNA of *CDR1as* with 43 sites. This circRNA-RBP interaction is also reported in a study of *hsa_circ_0001946* [23]. Taking all together, CircNetVis is not only able to recall well the interactions reported in previous studies but also provide a chance to explore other interactions from the networks.

## Conclusion

We have developed CircNetVis, an online web application for interactive visualization and investigation of interaction networks of circRNA. The interplays between circRNAs, miRNAs, mRNAs and RBPs are important to explore molecular functions of circRNAs and their signaling pathways.

### Supplementary Information

**Additional file 1.** Supplementary documents and figures.

## Data Availability

CircNetVis is implemented using R-shiny with external tools running in a Linux environment, and available at https://www.meb.ki.se/shiny/truvu/CircNetVis/. The source codes and data resources to run the web application are provided in its GitHub page at https://github.com/nghiavtr/CircNetVis.
