# Supplementary material for: CircNetVis: an interactive web application for visualizing interaction networks of circular RNAs
Source: BMC Bioinformatics. 2024 Jan 17;25:31. doi: 10.1186/s12859-024-05646-4 (PMC10795305; doi:10.1186/s12859-024-05646-4)
Supplement: Supplementary file 1 — Additional file 1. Supplementary documents and figures. [file 12859_2024_5646_MOESM1_ESM.docx]

Supplementary documents for “**CircNetVis: an interactive web application for visualizing interaction networks of circular RNAs**”

Thi-Hau Nguyen et al.

**Generation of circRNA sequences**

Given a circRNA ID with the start and stop coordinates, the sequence of this circRNA is generated using Circall-simulator (Nguyen *et al.*, 2021). For an exonic circRNA with less than three exons, its sequence is the concatenation of its exons. For an exonic circRNA with more than two exons, alternative splicing might occur which can produce more than one circRNA sequence. In this case, Circall-simulator generates all reasonable sequences of the circRNA based on the annotated transcriptome of the human genome reference hg19. The tool identifies all linear transcripts of the gene consisting of the start exon and the end exon of the circRNA. Then, all alternative splicing patterns in this region are extracted from the transcripts to generate the corresponding sequences of the circRNA. Furthermore, to capture the back-splicing junction region for the prediction of circRNA-miRNA interactions, the pseudo-sequence of a circRNA sequence is generated by adding L−1 last bases of circRNA sequence to the beginning of the circRNA sequence where L=28 equals to the maximum length of human microRNAs.

Thus, a circRNA ID might contain more than one pseudo-sequence due to alternative splicing (isoform). In practice, it is not straightforward to know which circRNA isoforms exist in the sample. Therefore, CircNetVis collects the predictions from the sequences of all circRNA isoforms, and the results of the best prediction among those circRNA isoforms are kept for the final report. It is noted that, if the exact sequence of the exonic circRNA is known, it can directly input into CircNetVis as the fasta format for interaction analyses.


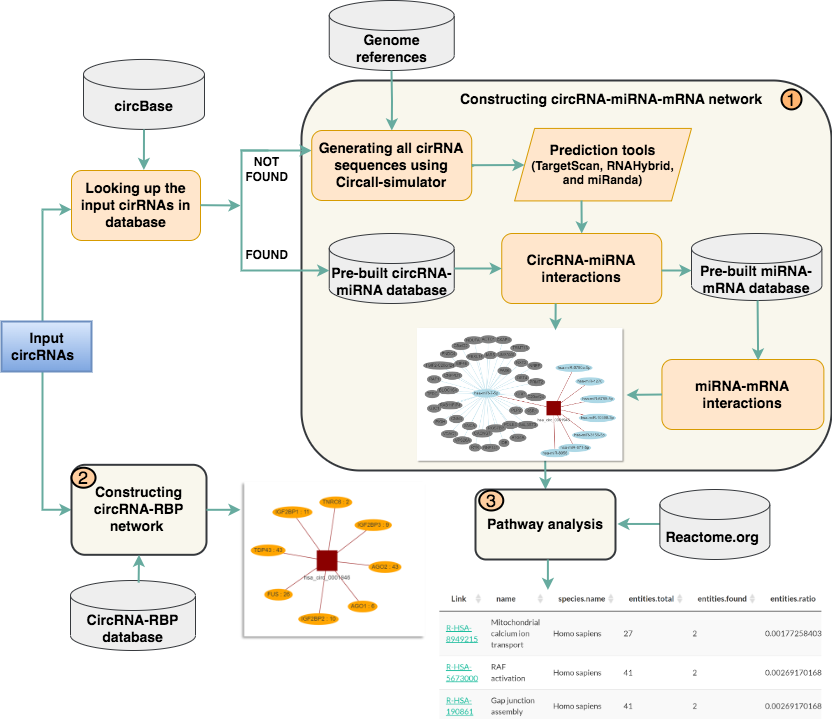


**Figure S1:** The workflow of CircNetVis in the backend including three main tasks: 1) construct circRNA-miRNA-mRNA network, 2) build circRNA-RBP network, and 3) pathway analysis. The circRNA-miRNA interactions are predicted using RNAhybrid, miRanda, and TargetScan. For all circRNAs existing the circBase, we predicted circRNA-miRNA interactions from the prediction tools and stored in a pre-built a database to reduce the workload for computation in the server of the web application. For new circRNAs, the prediction is performed directly on the server. The miRNA-mRNA interactions are collected from TargetScan database version 72; and the circRNA-RBP interactions are obtained from the CircInteractome database. The pathway analysis is performed using the gene set enrichment analysis of Reactome with the input from the list of the genes in the circRNA-miRNA-mRNA network*.*

**References**

Nguyen,D.T. *et al.* (2021) Circall: fast and accurate methodology for discovery of circular RNAs from paired-end RNA-sequencing data. *BMC Bioinformatics*, **22**, 495.
